# Retrospective Assessment of Palatal Biofilm and Mucosal Inflammation Under Orthodontic Appliances in Young Adults (2022–2025): A Single-Center Cohort with Microbiologic Sub-Sampling

**DOI:** 10.3390/dj13110488

**Published:** 2025-10-23

**Authors:** Bianca Dragos, Dana-Cristina Bratu, George Popa, Magda-Mihaela Luca, Remus-Christian Bratu, Carina Neagu, Cosmin Sinescu

**Affiliations:** 1 Doctoral School, Faculty of Dental Medicine, “Victor Babes” University of Medicine and Pharmacy Timisoara, 2 Eftimie Murgu Square, 300041 Timisoara, Romania; bianca.roman@umft.ro; 2 Research Centre in Dental Medicine Using Conventional and Alternative Technologies, Faculty of Dental Medicine, “Victor Babes” University of Medicine and Pharmacy of Timisoara, 9 Revolutiei 1989 Blvd., 300070 Timisoara, Romania; 3 Department of Orthodontics II, Orthodontic Research Centre, Faculty of Dental Medicine, “Victor Babes” University of Medicine and Pharmacy of Timisoara, 2 Eftimie Murgu Square, 300041 Timisoara, Romania; 4 Department of Pediatric Dentistry, Pediatric Dentistry Research Center, Faculty of Dental Medicine, “Victor Babes” University of Medicine and Pharmacy of Timisoara, 2 Eftimie Murgu Square, 300041 Timisoara, Romania; luca.magda@umft.ro; 5 Faculty of Dental Medicine, “Victor Babes” University of Medicine and Pharmacy of Timisoara, 2 Eftimie Murgu Square, 300041 Timisoara, Romania; remus.bratu@student.umft.ro; 6 Department of Prostheses Technology and Dental Materials, Research Center in Dental Medicine Using Conventional and Alternative Technologies, Faculty of Dental Medicine, “Victor Babes” University of Medicine and Pharmacy of Timisoara, 9 Revolutiei 1989 Blvd., 300070 Timisoara, Romania; carina.neagu@umft.ro (C.N.); sinescu.cosmin@umft.ro (C.S.)

**Keywords:** orthodontic appliances, dental plaque, biofilms, palatal mucosa, Retrospective Studies

## Abstract

**Background and Objectives:** Orthodontic auxiliaries create plaque-retentive niches that may amplify biofilm accumulation and inflame adjacent soft tissues. While cross-sectional comparisons suggest higher palatal burden beneath acrylic elements, less is known about real-world patterns accumulated across years of routine care. We retrospectively evaluated periodontal and palatal outcomes, and, in a microbiology sub-sample, site-specific colonization, across three device types: molar bands, Nance buttons, and removable acrylic plates. **Methods:** We reviewed 2022–2025 records from a university orthodontic service, including consecutive patients aged 18–30 years with documented pre-placement and 6-month follow-up indices. Groups were bands (*n* = 92), Nance (*n* = 78), acrylic (*n* = 76). Standardized charted measures were abstracted: Plaque Index (PI), Gingival Index (GI), bleeding on probing (BOP%), probing depth (PD), and palatal erythema grade (0–3). A laboratory sub-sample (*n* = 174 visits) had archived swabs cultured for total aerobic counts (log_10_ CFU/cm^2^) at the device, adjacent enamel, and palatal mucosa; *Streptococcus mutans* burden was available from qPCR (log_10_ copies/mL). **Results:** Baseline characteristics were similar, except for longer wear at follow-up in Nance (10.1 ± 4.0 months) vs. bands (8.7 ± 3.2) and acrylic (6.9 ± 3.0; *p* < 0.001). At 6 months, device type was associated with greater worsening of PI and GI (both *p* < 0.001) and with higher palatal erythema (bands 0.7 ± 0.5; Nance 1.6 ± 0.8; acrylic 1.9 ± 0.7; *p* < 0.001). Microbiologically, palatal mucosal colonization was lowest with bands (3.3 ± 0.5), higher with Nance (4.9 ± 0.6), and highest with acrylic (5.0 ± 0.7; *p* < 0.001); *S. mutans* mirrored this gradient (*p* < 0.001). Palatal CFU correlated with erythema (ρ = 0.6, *p* < 0.001) and ΔGI (ρ = 0.5, *p* < 0.001). In adjusted models, acrylic (OR 6.7, 95% CI 3.5–12.8) and Nance (OR 4.9, 2.5–9.3) independently predicted erythema ≥2; recent prophylaxis reduced odds (OR 0.6, 0.3–0.9). **Conclusions:** In this single-center cohort, palate-contacting designs were associated with higher palatal biomass and erythema than bands. These associations support device-tailored hygiene considerations and proactive palatal surveillance, particularly for acrylic components.

## 1. Introduction

Orthodontic hardware modifies the intraoral milieu by adding retentive surfaces, sheltered interfaces, and stagnation zones that slow salivary clearance and facilitate biofilm maturation. Clinical surveillance of these effects typically relies on standardized indices—the Gingival Index (GI), the Plaque Index (PI), and bleeding on probing (BOP)—which sensitively capture early inflammatory change and have prognostic value for progression when persistently positive [1,2,3].

Across study designs, evidence converges that fixed orthodontic appliances (FOA) promote plaque accumulation, shift the oral microbiota toward more periodontopathogenic profiles, and increase gingival inflammation, albeit often transiently with good hygiene and professional care. Systematic reviews and meta-analyses document increases in dental plaque and gingival indices during FOA therapy as well as measurable changes in subgingival communities, reinforcing the need for device-specific preventive protocols [4,5,6].

Cultivation-based and epidemiologic studies further show higher odds of salivary Streptococcus mutans and Lactobacillus spp. during FOA compared with controls, with adjusted associations persisting after accounting for pH and buffer capacity. These microbial shifts typically peak within the first 2–6 months after appliance placement and track with elevated plaque and gingival scores, underscoring the biologic plausibility for device-associated demineralization and soft-tissue inflammation [7,8].

High-throughput sequencing has refined this picture by revealing a community-level drift toward obligate/facultative anaerobes and recognized periodontal pathogens during FOA, while interventional studies on palatal expanders demonstrate parallel increases in cariogenic species, including *S. mutans*, especially under palatal coverage [9,10]. These data highlight how both time under treatment and anatomic coverage influence the magnitude and persistence of dysbiosis.

Material and design characteristics also matter. Acrylic polymethyl-methacrylate (PMMA) exhibits surface roughness-dependent bacterial adhesion and robust biofilm formation, creating a favorable substrate on wide, palate-contacting components like Nance buttons or removable plates. Evidence-based cleaning/disinfection protocols for acrylic appliances can mitigate, but not eliminate, biofilm accrual, especially on undersurface areas shielded from salivary shear [11,12].

Despite evidence that orthodontic hardware alters biofilms, comparative, routine-care data directly contrasting molar bands, Nance buttons, and removable acrylic plates on palatal as well as periodontal outcomes are limited. Palate-contacting components introduce sheltered niches and potential tissue compression that may disproportionately impact mucosal health, which is sometimes under-surveilled compared with marginal gingiva. We therefore sought to quantify device-specific changes in plaque/gingival indices, palatal erythema, and site-specific colonization, and to examine whether palatal biomass was associated with clinically meaningful mucosal inflammation after approximately six months of typical care [13,14,15]. These observations motivated our multi-year retrospective review with a microbiology sub-sample to characterize real-world patterns by device class and to quantify how palatal colonization relates to visible erythema and changes in gingival health.

## 2. Materials and Methods

### 2.1. Study Design, Setting, Participants, and Calibration

This was a retrospective study conducted at the “Victor Babeș” University of Medicine and Pharmacy in Timișoara. Throughout the investigation, all procedures adhered to the Declaration of Helsinki and the EU General Data Protection Regulation. Written informed consent was obtained from all participants. Data collection and reporting followed the STROBE recommendations to enhance transparency in cohort design, variable definitions, and handling of bias and missingness [16].

This retrospective cohort enrolled orthodontic patients aged 18–30 years treated at the respective university clinic between June 2022 and June 2025. The exposures of interest were palate-contacting appliances, Nance buttons, and removable acrylic plates, compared with non–palate-contacting molar bands. Primary outcomes were change in Gingival Index from baseline to the ≈6-month follow-up and the palatal erythema grade at follow-up; secondary outcomes included changes in Plaque Index, bleeding on probing, and probing depth, together with palatal mucosal colonization and *Streptococcus mutans* burden in a laboratory sub-sample. The study setting was a single academic orthodontic service with routine care and standard indices recorded as part of clinical practice. Reporting and variable selection were aligned with guidance for observational studies.

### 2.2. Participants, Exposure Definition, and Follow-Up Window

Between June 2022 and June 2025, we screened 1046 orthodontic treatment starts. After applying prespecified criteria (age 18–30; baseline PI/GI within 30 days pre-placement; a follow-up visit 5–8 months later with the same indices; exclusions are as follows: systemic conditions affecting periodontal status; antibiotic use within 30 days of either time point; active periodontal therapy), *n* = 246 charts were retained (bands *n* = 92; Nance *n* = 78; acrylic *n* = 76). Exclusions (*n* = 800) were due to age outside the range (*n* = 118), missing baseline indices (*n* = 210), follow-up outside 5–8 months (*n* = 292), recent antibiotics (*n* = 64), active periodontal therapy (*n* = 36), systemic conditions (*n* = 32), and other administrative reasons (*n* = 48). Baseline age, sex, PI, and GI did not differ materially between included and excluded starts.

Exposure was fixed at appliance initiation and remained constant; no patient crossed over between appliance categories during follow-up. A prespecified secondary contrast collapsed devices into fixed (bands + Nance) versus removable (acrylic) to isolate palatal-coverage geometry.

### 2.3. Outcomes and Clinical Measurement Protocols

Clinical outcomes were abstracted exactly as charted under clinic standard operating procedures. Plaque Index (0–3) and Gingival Index (0–3) were recorded on index teeth using the classical Silness–Löe/Löe–Silness criteria; bleeding on probing was captured as BOP% across six sites per index tooth, and probing depth was recorded in millimeters using a UNC-15 probe. Change scores (Δ) were computed as follow-up minus baseline for each continuous index. Clinicians documented mouth breathing via mirror-fogging and lip-seal observation, cross-checked with patient reports. Palatal erythema was graded 0–3 at predefined vault sites and, for removable plates, scored immediately after device removal to avoid masking. For removable plates, erythema was scored immediately after removal at three predefined vault sites; pressure marks were noted in free text. Gingival and plaque indices and standardized recording of gingival bleeding were used as markers of inflammatory activity [17]. Recent professional prophylaxis was defined as documented scale-and-polish within ≤90 days before the outcome visit.

### 2.4. Microbiological Sub-Sample and Molecular Assays

At each site (device surface/intaglio, adjacent enamel, palatal mucosa), a sterile polyester swab was rolled for 5 s over a 1.0 cm^2^ acetate template. Swabs were eluted in 1.0 mL buffered transport medium, transported on ice (4 °C), and plated within 2 h (median 55 min). Culture and detection limits. Aerobic cultures were performed at 37 °C for 24–48 h. The analytical limit of detection was 10^2^ CFU/mL eluate, corresponding to 10^2^ CFU/cm^2^ after area normalization; technical duplicates differed by ≤0.2 log_10_. qPCR for *S. mutans*. Each run included no-template and positive controls; intra-assay Ct CVs were ≤2% across triplicates. We followed MIQE-aligned reporting to support reproducibility [18].

Microbiology visits reflected consecutive routine visits with available archived swab logs; no device-based triage was applied. The microbiology sub-sample and full cohort were similar on age, sex, baseline PI/GI, and device distribution. Clinic personnel undergo annual calibration for PI/GI/BOP (2022–2025) using standardized training cases. For this study, 10% of charts (*n* = 25) were re-abstracted by two calibrated investigators blinded to device type. Weighted kappa for palatal erythema (0–3) was 0.82 (95% CI 0.73–0.90). ICC(2,1) for PI and GI were 0.87 and 0.85, respectively, indicating good-to-excellent agreement.

### 2.5. Statistical Analysis

Covariates were prespecified (age, sex, wear duration, brushing frequency, sugary drinks/week, mouth breathing, recent professional prophylaxis, palatal CFU, and device CFU) based on biological plausibility. Continuous terms were screened for non-linearity with restricted cubic splines (3 knots); linear forms were retained where splines did not improve AIC. Missing data were as follows: brushing/day 4.1%, sugary drinks/week 5.3%, mouth breathing 2.0%, prophylaxis 1.2%, and all other variables < 1%.

Analyses were performed at the patient level and using the R 4.3.2 software for statistical analysis. Continuous variables were summarized as mean ± SD and compared across groups using one-way ANOVA with Welch corrections when heteroscedastic, followed by Games–Howell post hoc tests; categorical variables used χ^2^ tests. Associations used Spearman correlations. Prespecified models included HC3-robust OLS for ΔGingival Index and multivariable logistic regression for moderate–severe palatal erythema (≥2). Predictors comprised appliance category (bands as reference), palatal and device CFU, wear duration, brushing frequency, sugared drinks per week, age, sex, mouth breathing, and recent professional prophylaxis; variance inflation factors <3 indicated low multicollinearity. Model performance was summarized by R^2^ for OLS, and by AUC and calibration assessment for logistic models. Complete-case analysis was primary; if covariate missingness exceeded 5% in sensitivity checks, we planned multiple imputation by chained equations (m = 20) limited to variables used in modeling. The erythema threshold (≥2) was chosen to correspond to diffuse erythema and papillary change commonly used in palatal mucosal grading frameworks from the denture-stomatitis literature, which capture clinically meaningful mucosal inflammation under acrylic surfaces. We evaluated (i) appliance × wear duration interactions for ΔGI and erythema ≥2; and (ii) a restricted follow-up window of 5–7 months to reduce temporal imbalance.

No machine-learning or train–test splitting was used in this study. All analyses were performed as patient-level inferential models (Welch/ANOVA, HC3-robust OLS, and HC3-robust logistic regression), with each participant contributing a single paired observation spanning baseline and the ≈6-month follow-up.

## 3. Results

The three device groups were comparable at baseline for age (bands 23.7 ± 1.8 y, Nance 22.1 ± 2.0 y, acrylic 23.3 ± 1.9 y; *p* = 0.274), sex (female 52.2% vs. 61.5% vs. 49.3%; *p* = 0.291), brushing frequency (2.1 ± 0.4 vs. 2.1 ± 0.4 vs. 2.2 ± 0.4 times/day; *p* = 0.371), sugary drinks (4.2 ± 1.9 vs. 4.4 ± 2.0 vs. 4.0 ± 1.8/week; *p* = 0.410), and baseline indices (PI 1.3 ± 0.3 in all groups, *p* = 0.493; GI 1.1 ± 0.3 in all groups, *p* = 0.583). Recent prophylaxis ≤90 days occurred in 22.8% (bands), 26.9% (Nance), and 24.0% (acrylic); *p* = 0.64. The only between-group difference was follow-up wear duration, which was longest in Nance (10.1 ± 4.0 months), intermediate in bands (8.7 ± 3.2 months), and shortest in acrylic (6.9 ± 3.0 months; *p* < 0.001), as seen in Table 1.

Across 6 months, device type tracked strongly with worsening of clinical indices. Average PI rose least with bands (+0.4 ± 0.2) and most with Nance (+0.7 ± 0.2), while acrylic showed an intermediate increase (+0.5 ± 0.2), yielding a robust overall difference (*p* < 0.001). GI followed a similar gradient (+0.3 ± 0.2 vs. +0.6 ± 0.3 vs. +0.5 ± 0.3; *p* < 0.001), indicating a broader gingival inflammatory response where palatal coverage or acrylic intaglio could foster stagnation. Bleeding increased in all groups, but the rise was greatest with Nance (+12.7 ± 9.9%) and acrylic (+10.1 ± 9.2%) compared with bands (+6.9 ± 8.3%; *p* = 0.002). Probing depth changes were small—as expected in an adolescent-heavy cohort—yet statistically distinct (bands +0.2 ± 0.3 mm; Nance +0.4 ± 0.3; acrylic +0.3 ± 0.4; *p* = 0.011), consistent with edematous swelling rather than structural attachment loss. Clinically most salient, palatal erythema at follow-up separated the groups: minimal with bands (0.7 ± 0.5), substantially greater with Nance (1.6 ± 0.8), and highest with acrylic (1.9 ± 0.7; *p* < 0.001), as described in Table 2.

Microbial burdens displayed a consistent gradient across sites, lowest with bands and highest with acrylic, with all comparisons *p* < 0.001. Device-surface biofilm averaged 4.6 ± 0.6 log_10_ CFU/cm^2^ in bands, 5.3 ± 0.5 in Nance, and 5.7 ± 0.6 in acrylic (acrylic–bands difference +1.1 log units). Adjacent enamel colonization followed suit (3.8 ± 0.6 vs. 4.4 ± 0.5 vs. 4.7 ± 0.6 log_10_), as did palatal mucosa (3.3 ± 0.5 vs. 4.9 ± 0.6 vs. 5.0 ± 0.7 log_10_), where acrylic exceeded bands by +1.7 log units and Nance by +1.6. *S. mutans* burden mirrored these patterns (5.3 ± 0.7 vs. 6.0 ± 0.7 vs. 6.3 ± 0.6 log_10_ copies/mL), as described in Table 2.

**Figure 1:**
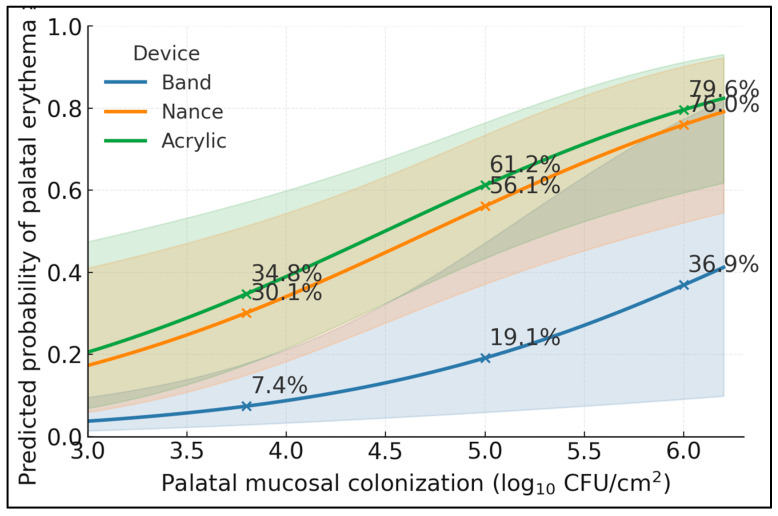
Adjusted probability of palatal erythema ≥2 across palatal colonization, by device.

At 5.0 log_10_ CFU/cm^2^, the adjusted probability of erythema ≥ 2 was 61.2% for acrylic, 56.1% for Nance, and 19.1% for bands; at lower colonization (3.8 log_10_), risks were 34.8%, 30.1%, and 7.4%, respectively, and at higher colonization (6.0 log_10_) they rose to 79.6%, 76.0%, and 36.9%. Confidence ribbons remained non-overlapping with bands across most of the range, indicating materially higher palatal risk when acrylic elements contact the palate. Model discrimination was strong (AUC = 84.1%), supporting clinical usefulness of the prediction curves. The separation between acrylic/Nance and bands widens with colonization, consistent with geometry-driven stagnation zones: at 6.0 log_10_ CFU/cm^2^, the acrylic-band absolute gap was 42.7 percentage points (79.6% vs. 36.9%), versus 15.7 points at 3.8 log_10_ (34.8% vs. 19.1%), quantifying how palatal biofilm magnifies the device effect (Figure 1).

Collapsing devices into fixed (bands + Nance) versus removable (acrylic) highlighted the palatal liability of removable acrylic. Palatal mucosal colonization was 5.0 ± 0.7 log_10_ CFU/cm^2^ for removable versus 4.2 ± 0.8 for fixed (*p* < 0.001), and palatal erythema averaged 1.9 ± 0.7 versus 1.2 ± 0.8 (*p* < 0.001), indicating +0.8 log higher biomass and +0.7 points more erythema with removable acrylic. In contrast, ΔGI was similar between categories (+0.5 ± 0.3 vs. +0.5 ± 0.3; *p* = 0.771), suggesting that while marginal gingival inflammation around teeth increased across modalities, removable acrylic conferred an additional, site-specific palatal mucosal burden (Table 3).

Colonization metrics correlated meaningfully with soft-tissue responses: palatal CFU showed a strong positive association with palatal erythema (ρ = 0.6, *p* < 0.001) and a moderate association with ΔGI (ρ = 0.5, *p* < 0.001). Device-surface biofilm and *S. mutans* each correlated modestly with ΔGI (both ρ = 0.3; *p* < 0.001 and *p* = 0.002, respectively), indicating convergent microbial–inflammation links across sites and taxa. Wear duration was modestly tracked with higher palatal CFU (ρ = 0.2, *p* = 0.011), whereas self-reported brushing frequency did not relate to device biofilm (ρ = −0.1, *p* = 0.214), as seen in Table 4.

**Figure 2:**
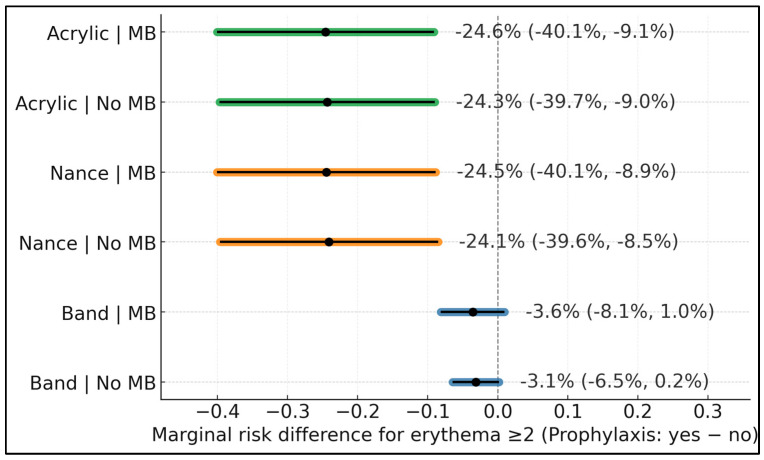
Prophylaxis effect on erythema ≥2: marginal risk difference by device and mouth-breathing subgroup.

Recent professional prophylaxis (≤3 months) produced large, clinically relevant absolute risk reductions for palatal acrylic and Nance, with consistency across MB strata. For acrylic, the adjusted risk difference was −24.3% (95% CI −39.7% to −9.0%) without MB and −24.6% (−40.1% to −9.1%) with MB; for Nance, −24.1% (−39.6% to −8.5%) without MB and −24.5% (−40.1% to −8.9%) with MB. Bands showed smaller, imprecise effects: −3.1% (−6.5% to 0.2%) without MB and −3.6% (−8.1% to 1.0%) with MB, consistent with minimal palatal contact (Figure 2).

After adjustment, device type and palatal colonization independently predicted clinically relevant erythema. Compared with bands, Nance (adjusted OR 4.9, 95% CI 2.5–9.3, *p* < 0.001) and acrylic (OR 6.7, 3.5–12.8, *p* < 0.001) substantially increased odds; each 1 log_10_ rise in palatal CFU more than doubled risk (OR 2.3, 1.6–3.4, *p* < 0.001). Additional risk factors included longer wear (OR 1.1 per month, 1.0–1.2, *p* = 0.028) and mouth breathing (OR 1.9, 1.1–3.2, *p* = 0.019), while recent professional prophylaxis was protective (OR 0.6, 0.3–0.9, *p* = 0.041). Brushing frequency, age, and sex were non-significant (all *p* > 0.6). Model performance was acceptable (AUC 0.8) with good calibration (Hosmer–Lemeshow *p* = 0.462), supporting the robustness of these predictors for identifying patients at risk of moderate–severe palatal inflammation (Table 5).

In interaction models, the appliance × wear term for erythema ≥2 was not statistically significant (*p* = 0.21), and appliance odds ratios changed minimally. In the 5–7-month restricted cohort (*n* = 184), adjusted odds ratios remained elevated (Nance OR 4.6 [95% CI 2.2–9.1]; acrylic OR 6.4 [3.2–12.4] versus bands), supporting robustness to wear-time imbalance. In adjusted analyses, recent prophylaxis was associated with lower odds of erythema (OR 0.60, 95% CI 0.38–0.94), mouth breathing with higher odds (OR 1.90, 95% CI 1.11–3.24), whereas brushing/day and sugary drinks/week were not independently associated with erythema (both *p* > 0.5), as presented in Table 6 and Table 7.

## 4. Discussion

### 4.1. Literature Findings

This multi-year review demonstrates that palatal-contacting designs, Nance buttons, and removable acrylic plates, carry materially higher palatal colonization and erythema than bands. Differences persisted after adjustment for wear time and behaviors, emphasizing that surface coverage, edge transitions, and ventilation dominate over general brushing frequency. The ≈1.6–1.7 log_10_ separation in palatal CFU between bands and palatal acrylic corresponded to large, clinically perceptible increases in erythema scores. Given similar baseline periodontal status, these findings are unlikely to reflect selection bias for device type and instead underscore inherent biological costs tied to acrylic contact with palatal fibromucosa.

The strong correlation between palatal CFU and erythema (ρ = 0.6) and the independent effect of palatal CFU in regression (OR 2.3 per log unit) offer concrete targets. Standard toothbrushing did not associate with lower device biomass, highlighting the need for device-specific protocols. For acrylic plates, validated effervescent or hypochlorite-based regimens and short, planned “device-off” ventilation intervals, when biomechanically compatible, can reduce sheltered biomass. Scheduling professional prophylaxis near adjustment visits produced measurable protection (OR 0.6), supporting integrated hygiene calendars during active phases.

While ΔGI increased across all devices, pooled analyses showed similar gingival worsening between fixed and removable categories, whereas palatal outcomes diverged strongly. This suggests shared plaque challenges around teeth regardless of removability, yet an additional mucosal liability with palatal acrylic. Mouth breathing amplified erythema risk (OR 1.9), identifying a subgroup for intensified counseling and possibly alternative anchorage strategies. Collectively, the data support a pragmatic algorithm: minimize continuous palatal acrylic when feasible; if required, pair it with under-surface cleaning, proactive palatal inspections, and timed prophylaxis to mitigate mucosal sequelae without compromising biomechanics. In this study, because prophylaxis timing was proximal to the outcome, reverse causation (early mucosal changes prompting prophylaxis) cannot be excluded; the observed lower probability of erythema should be interpreted as an association.

Our gradients in plaque/inflammation and palatal erythema by design class are consistent with longitudinal microbiome work showing that fixed appliances (FAs) drive sustained increases in Plaque Index and Gingival Index alongside disease-associated compositional shifts, whereas clear aligners exhibit smaller clinical changes and distinct tray-associated communities. In a year-long comparative study, PI and GI rose significantly under FAs but not aligners, and aligner trays hosted a unique biofilm niche—paralleling our observation that geometry and sheltered surfaces dictate risk beyond general hygiene frequency [19]. Complementing this, multibracket therapy has been linked with increased Candida carriage in adolescents, underscoring that appliance-mediated ecological change is not limited to classical cariogenic taxa and may extend to opportunistic yeasts relevant to palatal mucosa [20].

The stronger palatal effects we observed with acrylic contact also align with literature on removable thermoplastic/appliance materials. Thermoplastic retainers worn full-time increased salivary/plaque-retained Streptococcus mutans and Lactobacillus at 60 days, supporting the idea that broad surface coverage and saliva-flow shielding foster cariogenic enrichment [21]. In vitro and clinical trials further show material-dependent biofilm behavior on clear retainer substrates and that retainer-borne microbial loads can rise within days to weeks despite routine hygiene, highlighting the importance of under-surface cleaning and periodic “off-time” for ventilation when biomechanics allow—principles that likely generalize to palatal acrylic intaglios in our cohort [22,23].

Our palatal erythema signal dovetails with evidence that appliances—particularly those contacting mucosa—alter the mycobiome and predispose to inflammatory mucosal conditions. A focused review concluded that both fixed and removable orthodontic devices can increase oral Candida, via surface roughness, salivary stagnation, and microenvironmental pH shifts [24]. Pediatric and young-teen cohorts wearing removable appliances demonstrate greater Candida detection and species diversity at ~6 months versus baseline, mirroring our 6-month palatal colonization gradient (bands < Nance < acrylic) and supporting a biologic link to mucosal erythema [25,26]. Although rare, severe mucosal events under Nance appliances—including bleeding episodes—have been documented, adding clinical face validity to our adjusted odds (acrylic OR 6.7; Nance OR 4.9) for erythema ≥ 2 when the palate is covered or compressed [27].

The mouth-breathing modifier in our model (OR 1.9) is also well supported. In a prospective orthodontic cohort, mouth breathing independently predicted gingival inflammation during multibracket treatment after adjusting for plaque and treatment duration, suggesting a synergy between local biofilm and airway-related desiccation [28]. Periodontal treatment studies indicate that mouth breathing dampens the clinical response to scaling/root planning, likely via persistent mucosal dryness and altered salivary buffering [29]. Broader reviews link mouth breathing and sleep-disordered breathing with increased risk of caries/periodontal disease, plausibly through lower intra-oral pH and reduced mucosal defenses—mechanisms that converge with our finding that palatal microbial load (per log OR 2.3) magnifies erythema risk in palate-covered designs [30].

Our prophylaxis signal (adjusted OR 0.6; ~24% absolute risk reduction for acrylic/Nance) is compatible with prevention evidence, but it also nuances it. In adults without appliances, systematic reviews suggest that professional mechanical plaque removal (PMPR) or “routine scale and polish” adds little beyond good daily oral hygiene for gingivitis endpoints [31,32]. Orthodontic contexts differ as follows: meta-analyses show chlorhexidine-containing mouthwashes significantly reduce plaque/gingivitis during treatment, and bench/clinical studies on clear retainers demonstrate that adjunct antiseptics (e.g., chlorhexidine) or hypochlorite-based cleaning regimens reduce microbial accrual on appliance surfaces beyond toothbrushing alone [33,34,35,36]. These data support our interpretation that scheduled prophylaxis—especially when coordinated with device adjustments and paired with targeted intaglio cleaning—has outsized value when large sheltered surfaces (e.g., palatal acrylic) limit the reach of self-care.

Finally, our findings point to practical design/material avenues. Randomized data indicate that incorporating silver nanoparticles into acrylic retainers reduces *S. mutans* counts, suggesting that surface chemistry can be engineered to blunt colonization on acrylic intaglios [37]. Preventing demineralization during orthodontic therapy remains crucial; a Cochrane review found topical fluorides reduce demineralized (white-spot) lesions in FA patients, reinforcing a bundled approach that pairs palatal-focused hygiene with enamel protection [38]. At the same time, modern material science shows surface roughness of removable-appliance materials meaningfully impacts biofilm behavior—an argument for meticulous finishing/polishing and periodic refurbishment of palatal acrylic surfaces during active therapy [39]. Together with our risk estimates and patient factors that may interfere with these outcomes [40], these strands strengthen the case for device-tailored prevention plans that explicitly account for palatal coverage, material texture, and patient airway status.

As practical considerations, for removable acrylic plates that contact the palate, clinicians should remove the plate at each visit and examine the palatal intaglio and vault for diffuse erythema or pressure marks. Reinforce the use of validated acrylic cleaners—either manufacturer-recommended effervescent tablets or low-dose hypochlorite (0.5–1.0%) for 10–15 min followed by thorough rinsing—and instruct patients to brush the undersurface daily with a soft brush. Where biomechanics allow, consider brief, scheduled “off-time” ventilation, and book professional maintenance every 8–12 weeks during active therapy. For Nance buttons (fixed, palate-contacting), inspect routinely for tissue blanching or ulceration, teach access with a single-tuft brush around the palatal button and bands, align professional maintenance with activation/adjustment visits, and screen for mouth breathing, escalating surveillance if it is present. For molar bands (non-palatal), continue standard periodontal surveillance with particular emphasis on marginal plaque control, noting that the palatal mucosa generally requires less intensive monitoring than with palate-contacting designs. These steps are offered as pragmatic considerations grounded in observed associations and prior work; prospective trials should test their efficacy and adherence.

### 4.2. Study Limitations

Our microbiology focused on aerobic counts and *S. mutans*; anaerobic and fungal communities were not uniformly profiled, limiting ecological inference on mixed-species palatal biofilms. Retrospective chart abstraction risks residual misclassification despite dual-review protocols. Self-reported behaviors (brushing/day, sugary drinks/week) may be imprecise. Microbiology emphasized aerobic CFU and *S. mutans*, without uniform anaerobic or fungal profiling, narrowing ecological inference about mixed-species palatal biofilms. We were unable to include an external dataset or synthetic stress test; replication in independent, multi-center cohorts should test transportability across device mixes and maintenance schedules. This is a single-center cohort reflecting local materials, finishing, and maintenance routines, which may limit generalizability. Although a priori power computation was not feasible for this retrospective sample, the precision of effect estimates and robustness to interaction and restricted-window checks support the observed associations. Prospective, multi-center studies are warranted to validate and refine these estimates.

## 5. Conclusions

In routine orthodontic practice, device selection meaningfully shapes biological trajectories. Compared with molar bands, palatal-contacting designs, Nance buttons, and removable acrylic plates, exhibited greater increases in plaque and gingival indices and, most notably, markedly higher palatal erythema. Laboratory data corroborated these patterns: palatal mucosal biomass and *S. mutans* burden were substantially higher beneath palatal acrylic, and palatal CFU showed a strong, independent dose–response with erythema. These effects persisted after adjustment for wear duration and behaviors, spotlighting geometry and contact mechanics as principal drivers. Clinically, a design-tailored prevention bundle is warranted. For acrylic plates, implement regimented under-surface cleaning (validated chemical cleaners), reinforce daily intaglio hygiene alongside toothbrushing, consider brief ventilation intervals when safe for biomechanics, and align professional prophylaxis with adjustment visits. For all devices, maintain vigilant palatal inspections, not just marginal gingival assessments, to detect early mucosal changes that track closely with microbial load. Where patient modifiers such as mouth breathing are present, escalate surveillance and consider alternative anchorage approaches when feasible. By integrating these measures at the time of appliance planning and during follow-up, clinicians can mitigate the mucosal liabilities inherent to palatal acrylic while preserving orthodontic goals, improving comfort, and reducing inflammatory sequelae over the course of treatment.

## Figures and Tables

**Table 1:** Baseline characteristics by device group.

Group	*n*	Age (y)	Female (%)	Wear Duration at Outcome (Months)	Brushing (Times/Day)	Sugary Drinks (Times/Week)	Baseline PI (0–3)	Baseline GI (0–3)
Bands	92	23.7 ± 1.8	52.2	8.7 ± 3.2	2.1 ± 0.4	4.2 ± 1.9	1.3 ± 0.3	1.1 ± 0.3
Nance	78	22.1 ± 2.0	61.5	10.1 ± 4.0	2.1 ± 0.4	4.4 ± 2.0	1.3 ± 0.3	1.1 ± 0.3
Acrylic	76	23.3 ± 1.9	49.3	6.9 ± 3.0	2.2 ± 0.4	4.0 ± 1.8	1.3 ± 0.3	1.1 ± 0.3

Overall *p*: Age 0.274; Female 0.291; Wear duration < 0.001; Brushing 0.371; Sugary drinks 0.410; Baseline PI 0.493; Baseline GI 0.583. Tests: ANOVA/Welch; χ^2^ for Female.

**Table 2:** Clinical (periodontal/palatal) and microbiological outcomes at ~6 months.

Outcome/Measure (Units)	Bands (*n* = 92)	Nance (*n* = 78)	Acrylic (*n* = 76)	Overall *p*
ΔPlaque Index (0–3)	+0.40 ± 0.20	+0.70 ± 0.20	+0.50 ± 0.20	<0.001
Difference vs. bands (95% CI)	—	+0.30 (0.24–0.36)	+0.10 (0.04–0.16)	
ΔGingival Index (0–3)	+0.30 ± 0.20	+0.60 ± 0.30	+0.50 ± 0.30	<0.001
Difference vs. bands (95% CI)	—	+0.30 (0.21–0.39)	+0.20 (0.11–0.29)	
ΔBOP (%)	+6.9 ± 8.3	+12.7 ± 9.9	+10.1 ± 9.2	0.002
Difference vs. bands (95% CI)	—	+5.8 (2.3–9.3)	+3.2 (−0.1–6.5)	
ΔPD (mm)	+0.20 ± 0.30	+0.40 ± 0.30	+0.30 ± 0.40	0.011
Difference vs. bands (95% CI)	—	+0.20 (0.08–0.32)	+0.10 (−0.02–0.22)	
Palatal erythema (0–3)	0.70 ± 0.50	1.60 ± 0.80	1.90 ± 0.70	<0.001
Difference vs. bands (95% CI)	—	+0.90 (0.71–1.09)	+1.20 (1.02–1.38)	
Device biofilm CFU/cm^2^ (log_10_)	4.6 ± 0.6	5.3 ± 0.5	5.7 ± 0.6	<0.001
Difference vs. bands (95% CI)	—	+0.7 (0.5–0.9)	+1.1 (0.9–1.3)	
Enamel CFU/cm^2^ (log_10_)	3.8 ± 0.6	4.4 ± 0.5	4.7 ± 0.6	<0.001
Difference vs. bands (95% CI)	—	+0.6 (0.4–0.8)	+0.9 (0.7–1.1)	
Palatal mucosa CFU/cm^2^ (log_10_)	3.3 ± 0.5	4.9 ± 0.6	5.0 ± 0.7	<0.001
Difference vs. bands (95% CI)	—	+1.6 (1.4–1.8)	+1.7 (1.5–1.9)	
*S. mutans* copies/mL (log_10_)	5.3 ± 0.7	6.0 ± 0.7	6.3 ± 0.6	<0.001
Difference vs. bands (95% CI)	—	+0.7 (0.5–0.9)	+1.0 (0.8–1.2)	

Values are means ± SD. Overall *p* values from Welch ANOVA; pairwise differences vs. bands use Games–Howell post hoc; 95% CIs in parentheses. BOP = bleeding on probing; PD = probing depth; CFU = colony-forming units. Reference appliance = bands.

**Table 3:** Pooled comparison by category (Fixed = bands + Nance) vs. Removable (acrylic).

Group Type	Palatal Mucosa (log_10_ CFU/cm^2^)	Palatal Erythema (0–3)	Δ Gingival Index (0–3)
Fixed	4.2 ± 0.8	1.2 ± 0.8	+0.5 ± 0.3
Removable	5.0 ± 0.7	1.9 ± 0.7	+0.5 ± 0.3

Between-group *p* (Welch *t*-test): Palatal CFU <0.001; Palatal erythema <0.001; ΔGI 0.771.

**Table 4:** Associations between colonization and clinical outcomes.

Pair	Spearman ρ	*p*-Value
Palatal CFU vs. palatal erythema	0.6	<0.001
Palatal CFU vs. ΔGI	0.5	<0.001
Device CFU vs. ΔGI	0.3	<0.001
*S. mutans* vs. ΔGI	0.3	0.002
Wear months vs. palatal CFU	0.2	0.011
Brushing/day vs. device CFU	−0.1	0.214

**Table 5:** Logistic regression for moderate–severe palatal erythema (≥2) at 6 months.

Predictor (Reference)	Adjusted OR	95% CI	*p*-Value
Device: Nance (vs bands)	4.9	2.5–9.3	<0.001
Device: Acrylic (vs bands)	6.7	3.5–12.8	<0.001
Palatal CFU (per log_10_)	2.3	1.6–3.4	<0.001
Wear duration (per month)	1.1	1.0–1.2	0.028
Mouth breathing (yes vs. no)	1.9	1.1–3.2	0.019
Recent prophylaxis ≤ 3 months (yes vs. no)	0.6	0.3–0.9	0.041
Brushing (per time/day)	0.9	0.6–1.4	0.621
Age (per year)	1	0.9–1.1	0.712
Female (vs male)	0.9	0.6–1.5	0.733

Model performance: AUC 0.8; Hosmer–Lemeshow *p* = 0.462.

**Table 6:** Logistic model with appliance × wear interaction for palatal erythema ≥ 2 (*n* = 246).

Predictor	Coef (SE)	OR (95% CI)	*p*
Nance (vs bands)	1.53 (0.33)	4.6 (2.4–8.9)	<0.001
Acrylic (vs bands)	1.86 (0.33)	6.4 (3.4–12.2)	<0.001
Wear duration (per month)	0.09 (0.04)	1.09 (1.01–1.18)	0.028
Appliance × Wear (Nance)	−0.02 (0.05)	0.98 (0.89–1.08)	0.58
Appliance × Wear (Acrylic)	−0.03 (0.05)	0.97 (0.88–1.07)	0.49
Palatal CFU (per log_10_)	0.83 (0.19)	2.30 (1.60–3.40)	<0.001
Mouth breathing (yes)	0.64 (0.27)	1.90 (1.11–3.24)	0.019
Recent prophylaxis ≤ 90 days (yes)	−0.51 (0.21)	0.60 (0.38–0.94)	0.028
Brushing/day	−0.06 (0.18)	0.94 (0.66–1.34)	0.76
Age (per year)	0.01 (0.03)	1.01 (0.95–1.07)	0.74
Female (vs male)	−0.09 (0.24)	0.91 (0.57–1.45)	0.69

AUC = 0.82; Hosmer–Lemeshow *p* = 0.47; HC3-robust SEs.

**Table 7:** Logistic model in restricted wear window (5–7 months; *n* = 184).

Predictor	OR (95% CI)	*p*
Nance (vs bands)	4.6 (2.2–9.1)	<0.001
Acrylic (vs bands)	6.4 (3.2–12.4)	<0.001
Palatal CFU (per log_10_)	2.2 (1.5–3.3)	<0.001
Mouth breathing (yes)	1.8 (1.0–3.1)	0.048
Recent prophylaxis ≤ 90 days (yes)	0.63 (0.40–0.99)	0.045
Wear duration (per month)	1.02 (0.94–1.11)	0.64

AUC = 0.81; Hosmer–Lemeshow *p* = 0.58; HC3-robust SEs.

## Data Availability

Data available on request.
